# Hypomania-Checklist-33: risk stratification and factor structure in a mixed psychiatric adolescent sample

**DOI:** 10.1186/s40345-024-00350-x

**Published:** 2024-08-07

**Authors:** Miriam Gerstenberg, Lukasz Smigielski, Anna M. Werling, Maria E. Dimitriades, Christoph U. Correll, Susanne Walitza, Jules Angst

**Affiliations:** 1 Department of Child and Adolescent Psychiatry and Psychotherapy, Psychiatric University Hospital Zurich, University of Zurich, Zurich, Switzerland; 2 Child Development Center, University Children’s Hospital Zurich, Zurich, Switzerland; 3 Department of Psychiatry, The Zucker Hillside Hospital, Northwell Health, Glen Oaks, NY USA; 4 Department of Psychiatry and Molecular Medicine, Donald and Barbara Zucker School of Medicine at Hofstra/Northwell, Hempstead, NY USA; 5 Department of Child and Adolescent Psychiatry, Charité-Universitätsmedizin Berlin, Berlin, Germany; 6 German Center for Mental Health (DZPG), Partner Site Berlin, Berlin, Germany; 7 University of Zurich, Zurich, Switzerland; 8 Department of Child and Adolescent Psychiatry and Psychotherapy, Outpatient Services Winterthur, Psychiatric University Hospital Zurich, Albanistrasse 24, 8400 Winterthur, Switzerland

**Keywords:** Hypomania, Depression, Adolescence, Bipolar disorder, At-risk for bipolar disorder, HCL-33

## Abstract

**Background:**

The 33-item Hypomania Checklist (HCL-33) has been shown to distinguish between adolescent bipolar disorder (BD) and unipolar depression. To investigate the utility of the HCL-33 as a screening tool in routine diagnostics, the frequency and psychopathological characteristics of detected individuals in a mixed psychiatric sample necessitate more examination.

**Methods:**

The HCL-33, Children’s Depression Inventory, Beck’s Anxiety Inventory, and Strengths and Difficulties Questionnaire were completed by 285 children and adolescents (12–18 years) in a mixed psychiatric sample. Applying the proposed HCL-33 cut-off score of ≥ 18, individuals with depressive symptoms were divided into at-risk or not at-risk for BD groups. The factorial structure, sum and factor score correlations with psychopathology, and impact on daily functioning were assessed.

**Results:**

20.6% of the sample met at-risk criteria for BD. These individuals (*n* = 55) were older, more anxious, and showed more conduct problems vs the not at-risk group (*n* = 107). A two- and a three-factor model were pursued with the same Factor 1 (“active-elated”). Factor 2 (“risk-taking/irritable”) was separated into 2a (“irritable-erratic”) and 2b (“outgoing-disinhibited”) in the three-factor model. Whereas higher Factor 2 and 2a scores correlated with a broad range of more severe symptomatology (i.e., depression, anxiety, hyperactivity), higher Factor 1 and 2b scores correlated with more emotional and conduct problems, respectively. 51.7% of the sample reported a negative impact from hypomanic symptoms on daily functioning.

**Limitations:**

Cross-sectional design and data collection in a single mental health service.

**Conclusions:**

The HCL-33 may be a useful tool to improve diagnostics, especially in adolescents with depressive symptoms additionally presenting with anxious symptoms and conduct problems.

**Supplementary Information:**

The online version contains supplementary material available at 10.1186/s40345-024-00350-x.

## Introduction

Bipolar disorder (BD), which is characterized by alternating episodes of depression and mania or hypomania, is one of the largest contributors to disability-adjusted life years in young people (Crump et al. 2013; Ferrari et al. 2016; McIntyre et al. 2020). Depressive episodes increase in prevalence from childhood to adolescence, and an earlier onset age of major depression disorder (MDD) is hypothesized to be a risk factor for developing BD (Akiskal 1995; Geller et al. 1994; Hauser and Correll 2013; Malhi et al. 2020; Merikangas et al. 2009; Rao et al. 1995; Tondo et al. 2010). The incidence of manic or hypomanic (hereafter summarized and referred to as “(hypo)manic”) episodes peaks around age 17 years (Baldessarini et al. 2012; Bechdolf et al. 2012a; Kessler et al. 2012). Thus, late childhood and adolescence represent developmental phases during which increased efforts to identify BD are crucial (Correll et al. 2007a; Fritz et al. 2017; Hauser and Correll 2013; Hauser et al. 2007).

Recent meta-analyses indicate that in child and adolescent populations, 1.8% have diagnosed BD and 3.9% have bipolar-spectrum disorders (Meter et al. 2019). Bipolar-spectrum disorders are more common than schizophrenia or autism-spectrum disorders, and less common than MDD or attention-deficit/hyperactivity disorder (ADHD) (Costello et al. 2005; Goldstein et al. 2017). Nevertheless, it is suspected that pediatric BD is underdiagnosed or frequently misdiagnosed (Goldstein et al. 2017; Weller et al. 1995).

Maturational processes, highly dynamic behavioural changes, varying symptom presentation, and high rates of comorbidities may all contribute to a delay in detection of (hypo)manic episodes in young populations (Geller and Luby 1997; Kessler et al. 2012; Tillman and Geller 2003; Youngstorm et al. 2008). Specifically, core symptoms of a (hypo)manic episode, such as abnormal levels of irritability and activity, or elated mood, may also occur during typical adolescence and with other psychiatric disorders, such as ADHD, anxiety disorders, and substance use disorders ( Brand et al. 2010; Geller et al. 2000; Tillman and Geller 2003). Thus, episodic occurrence and/or clustering of (hypo)manic symptoms may be masked by continuous symptoms of comorbidities, which further complicates the early detection of BD. Further, as recently there has been an exponential increase in information regarding the phenotype, frequency, and treatment of BD, it is possible that some senior clinicans who completed their training earlier are less likely to encourage the screening for and monitoring of (hypo)manic symptoms in children and adolescents (Goldstein et al. 2017). Indeed, a recent longitudinal register study underlines that children and adolescents attending child and adolescent mental health services have an overall elevated risk of a later diagnosis of BD (Lång et al. 2022). Assessing the predictive power of specific diagnoses made during the first 3 months of contact with these specialized services, the highest risk for a BD diagnosis during the following years until age 28 was reported for depressive or other mood disorders (Lång et al. 2022). These findings emphasize the potential of early detection efforts within these specialized services in unselected clinical samples as well as in individuals presenting with depressive symptoms (Correll et al. 2007a; Lång et al. 2022).

In everyday clinical practice, screening instruments may critically improve the detection of symptoms, paving the way for in-depth diagnostics and targeted interventions (Carta and Angst 2016; Goldstein et al. 2017; Youngstrom et al. 2015). Early treatments ameliorate functional outcomes, whereas a longer duration of untreated illness is associated with worse outcomes (Altamura et al. 2010; Berk et al. 2011; Post et al. 2010). As younger age of BD onset is associated with more frequent and severe manic episodes, a longer delay in first treatment, and suicidal behaviour, the need for early detection, especially in children and adolescents, is of critical importance (Cate Carter et al. 2003; Correll et al. 2007b; Faedda et al. 2019; Joslyn et al. 2016; Meter et al. 2016; Post et al. 2010; Perlis et al. 2004; Pfennig et al. 2020).

The 32-item Hypomania Check List-32 (HCL-32) was originally developed as a self-report screening tool for past (hypo)manic symptoms in adult patients affected by MDD (Angst et al. 2005; Forty et al. 2009). In adults, the HCL-32 has been intensively studied and suggested as a sensitive and useful screening instrument in the context of patients with mood disorders and more generally, in routine psychiatric care (Meyer et al. 2011; Woo and Crowell 2005). To date, the HCL-32 and its slightly adapted successor version, the 33-item Hypomania Checklist (HCL-33), have not been thoroughly assessed in a child and adolescent mixed psychiatric sample. The HCL-33 combines two items regarding sexual desire and activity into one item and integrates two additional questions on gaming and binge eating behaviour (Feng et al. 2016). One study introduced the HCL-33 in adolescents diagnosed with MDD or BD and found that 18 affirmative responses was the optimal cut-off value to best distinguish between the two disorders (Zhang et al. 2021). However, there is still a knowledge gap regarding the frequency of (hypo)manic symptoms and associations with other psychopathology in children and adolescents, which may contribute to the difficulties diagnosing and treating these individuals.

To uncover the different symptom dimensions of (hypo)mania identified in the structure of the self-report questionnaire, factor analytical approaches were applied. Most studies in adults indicated that the HCL-32 and HCL-33 items map onto two latent constructs, or factors, in clinical and non-clinical samples (Angst et al. 2005, 2010; Feng et al. 2016; Gamma et al. 2013; Meyer et al. 2007; Zhang et al. 2021). In this two-factor structure, Factor 1 (“active-elated”) represents positively connotated questions, and Factor 2 (“risk-taking/irritable”) represents negatively connotated questions. In one non-clinical adolescent sample, HCL-32 Factor 1 was similar to adults, whereas Factor 2 seemed to be better reflected by two separate factors, Factor 2a (“disinhibited/stimulation-seeking”) and Factor 2b (“irritable-erratic”). Factors 2a and 2b were associated with more conduct problems, and Factor 2b was additionally linked to increased hyperactivity-inattention and social issues. Therefore, it has been hypothesized that the HCL-32 Factors 2a and 2b better represent age-specific features of BD (Holtmann et al. 2009).

Taken together, the identification of individuals with elevated risk for developing BD, and characterization of (hypo)manic symptom dimensions are relevant for diagnostic efforts and early detection in young individuals with different mental health disorders. Thus, the first objective of this study was to determine the frequency, demographic, and diagnostic correlates of past (hypo)manic symptoms in 12–18-year-old individuals from a mixed psychiatric sample. Next, we focussed on individuals presenting with depressive symptoms as a group enriched in risk for missed as well as subsequent BD diagnoses. In these individuals, we applied the proposed cut-off score ≥ 18 for the HCL-33 to investigate differences between the at-risk (above HCL-threshold) and not at-risk groups for BD (below HCL-threshold). Lastly, we assessed the factorial structure of the HCL-33 and sum/factor score associations with current psychopathology and negative impact on daily life functioning.

## Methods

### Design and procedures

This retrospective clinical chart review study using routine clinical care data was in accordance with the Declaration of Helsinki and approved by the local ethics committee of Zurich (KEK-ZH 2015-0065). In this study, the use of health-related personal data without informed consent procedures were permitted under the application of the article 34 of the Human Research Act.

### Participants

Participants were included who were (1) aged 12–18 years, and (2) assessed and/or treated during a 2-year interval through inpatient and outpatient mental health services at the Department of Child and Adolescent Psychiatry and Psychotherapy, Psychiatric University Hospital Zurich, Switzerland. Young individuals (*n* = 285) completed the HCL-33 as part of routine clinical baseline assessments alongside other questionnaires. Individuals were assessed in a multidisciplinary setting by psychiatrists and psychologists with special training in child and adolescent psychiatry and psychotherapy and on multiple occasions before an ICD-10 or no diagnosis was confirmed by a senior child and adolescent psychiatry specialist. In this analysis, confirmed clinical diagnoses within 4 months of administration of the questionnaire were considered and treatment setting at the time of the assessment was derived from clinical chart information. Total intelligence quotient scores were obtained, if assessed, with the Wechsler Intelligence Scale for Children-Fourth Edition or Wechsler Adult Intelligence Scale (Kaufman 2006; Laney et al. 2011).

### Questionnaires

The HCL-33 is a brief, self-report, developed to screen for lifetime (hypo)manic symptoms in adults (Feng et al. 2016). The HCL-33 is comprised of 33 questions targeting (hypo)manic symptoms. All 33 items require a yes/no response. All affirmative answers (‘yes’) are summed to calculate the total score ranging from 0 to 33. The questionnaire also includes additional questions pertaining to the impact of (hypo)manic episodes on four domains of daily life (family life, social life, work/school, leisure). In this study, we focussed on the 33 single items and the additional questions assessing the reported impact on daily life. There were four options to specify the impact of lifetime (hypo)manic symptoms on the respective domain of daily life: (1) positive and negative impact, (2) positive impact, (3) negative impact, and/or (4) no impact. As positive impact by itself is rarely reported when assessing psychiatric symptoms, the project focussed on negative impact, encoded as: as (1) = 1, (2) = 0, (3) = 1, and (4) = 0.

The German version of the Depression Inventory for Children and Adolescents (CDI) was used to screen for and rate the severity of recent depressive symptoms (Kovacs 1992; Stiensmeier-Pelster et al. 2000). The CDI is comprised of 26 questions, each of which is answered on a scale from “0 = symptom not present” to “2 = symptom strongly pronounced”. A total score was derived by summing all ratings.

The Beck Anxiety Inventory (BAI) is a 21-item inventory that measures the severity of anxiety during the past week. Twenty-one symptoms of anxiety are rated on a scale from “0 = not present” to “3 = strongly present”. Individual items are summed to obtain a total score (Beck et al. 1988).

The Strengths and Difficulties Questionnaire (SDQ) screens for general psychopathological impairments during the past 6 months (Goodman 1997). It is comprised of 25 items, with five subscales of five questions each, i.e., emotional symptoms, behavioural problems, hyperactivity/inattention, peer problems, but also prosocial behaviour. Each item is scored on a scale from “0 = not true” to “2 = certainly true”. A total score, ranging from 0 to 40, is obtained by summing all items except for the prosocial score.

### Risk grouping

We focussed on young individuals with a depression severity score of ≥ 17 according to the CDI. This cut-off was previously shown to maximize sensitivity (0.81) and specificity (0.84) in individuals diagnosed with MDD in a mixed psychiatric inpatient sample (Craighead et al. 1995; Kovacs and Pollock 1995). This focus was chosen, because the risk of missed (hypo)manic symptoms during the lifetime may be increased especially in those individuals (Akiskal 1995; Fritz et al. 2017; Mesman et al. 2017). Further, the HCL was originally developed to detect past (hypo)manic symptoms in adult patients affected with MDD (Angst et al. 2005; Forty et al. 2009).

Individuals meeting the CDI cut-off were further grouped into at-risk and not at-risk for BD according to the previously published cut-off score of ≥ 18 affirmative responses in the HCL-33 (Zhang et al. 2021).

### Data analysis

Statistical analyses were performed using SPSS® 26.0 for Windows software (IBM Corp., Armonk, NY, USA) and with the R software environment for statistical computing version 4.1.0 (for exploratory factor analysis). Descriptive statistics were reported for sociodemographic and diagnostic characteristics and for questionnaire scores as means ± standard deviation (SD). To examine possible differences between male and female participants, the Mann–Whitney-U test and chi-square test were used.

Groups were compared using group-specific descriptive statistics. The single-item analysis was performed using a chi-square test. Differences on the single-item level were controlled for multiple comparison using Bonferroni correction.

**Fig. 1:**
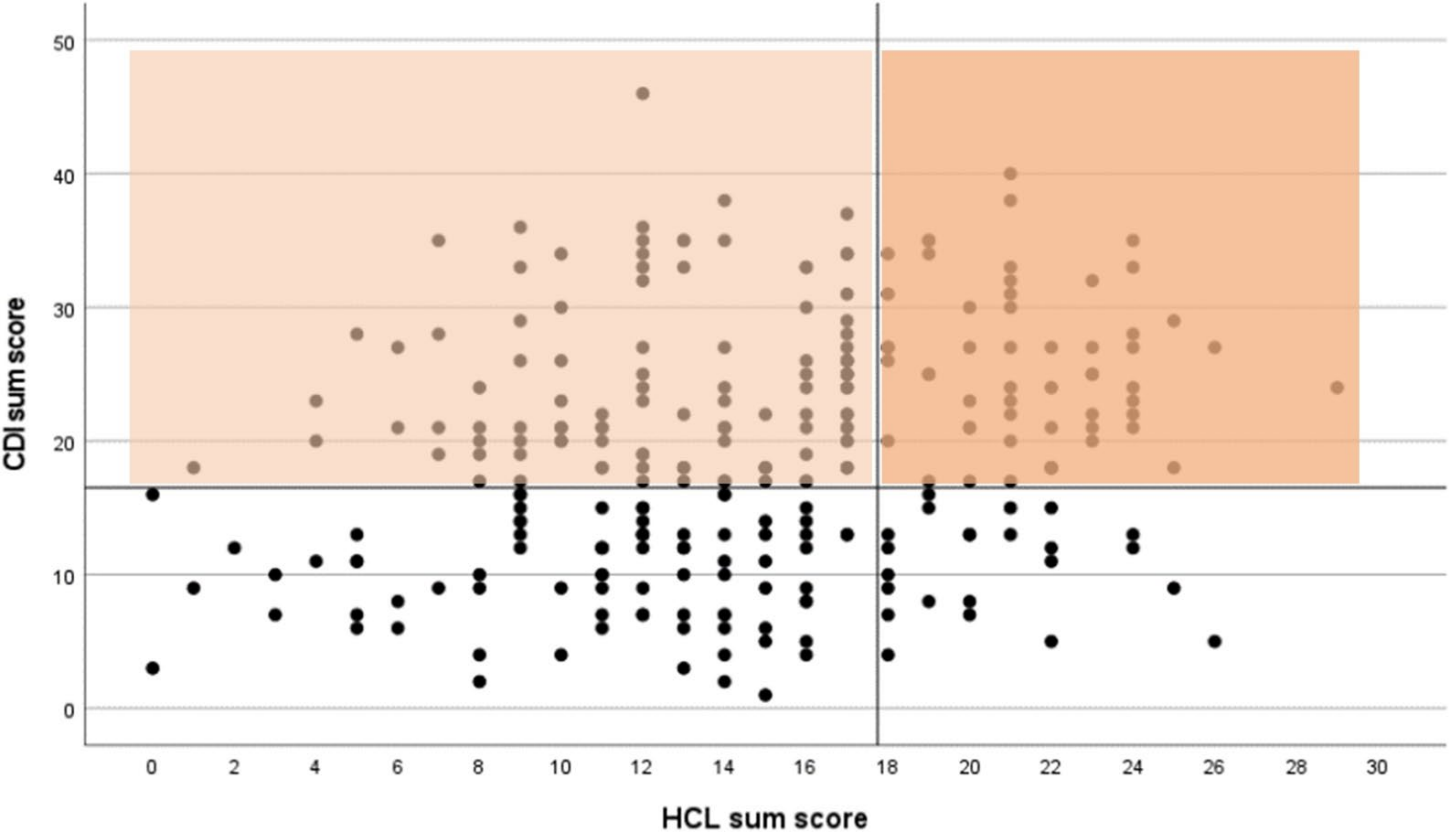
Scatterplot of Hypomania Checklist-33 sum score vs. Children’s Depression Inventory sum score. Dots represent the individual sum scores according to the Hypomania Checklist-33 (HCL-33) (x-axis) and Children’s Depression Inventory (CDI) (y-axis). The horizontal line where the CDI sum score equals 17, represents the respective threshold. For further analysis, all individuals reporting CDI sum scores ≥ 17 (current depression) were considered and individuals below this threshold (no current depression) were excluded. The vertical line where the HCL sum score equals 18, represents the respective threshold. The brighter colored left sector comprises individuals with depressive symptoms below the HCL threshold (“depressed not at-risk for BD”, *n* = 107). The darker colored right sector comprises individuals with current depression also reporting HCL sum scores ≥ 18 (“depressed at-risk for BD, *n* = 55)

An exploratory factor analysis (EFA) was conducted to assess the underlying dimensionality structure of the HCL-33 using the subsequent steps, as instructed in the ‘psych’ R package (https://cran.r-project.org/web/packages/psych/index.html). Data adequacy for factor analysis was statistically inspected prior to the procedure. Given the binary response format, tetrachoric correlations were calculated for the 33 dichotomous items (Fig. 1S). Oblique factor rotation was applied in this analysis. The number of factors to retain was decided based on several criteria to optimize the final solution. First, a scree plot was used to visualize the magnitude of the successive eigenvalues and inspect the point at which they plateau in a graphical representation (Cattell 1966). Second, the parallel analysis approach was applied to extract and compare factor solutions of real data and of data with the same properties but generated by simulation (*fa.parallel* algorithm) (Fig. 3S) (Hayton et al. 2004; Horn 1965). The candidate factors are indicated in this method by their eigenvalues being larger than their randomized counterparts. Third, the Very Simple Structure (VSS) method was used to evaluate a range of solutions of increasing rank complexity by estimating the top-loading item and restricting factor loading to zero (Fig. 5S) (Revelle and Rocklin 1979). The candidate factors are indicated in this method by the maximized fit to the observed correlation matrix. Finally, clinical considerations regarding the coherence and interpretability of the factors were considered. Items were assigned to a specific factor when their loadings were at least 0.4, mirroring the most common threshold reported in the literature (Howard 2016). The derived factors were inspected for reliability using Cronbach’s alpha. The association of HCL-33 sum and factor scores with demographic characteristics was assessed. Pearson’s correlation coefficients were used to investigate associations between the HCL-33 sum scores, factors, and psychopathology measures. When required, the correlation analysis was adjusted for confounding demographic variables.

Finally, the frequency of individuals who reported a negative impact from (hypo)manic symptoms on one or more of the four domains of daily life (family life, social life, work/school, leisure) was investigated. For each of the four domains of daily life, two groups were formed: (1) individuals who reported negative impact, and (2) individuals who reported no negative impact on the respective domain of daily life. Their HCL-sum and factor scores were compared using Mann-Whithney U tests. All tests were two-sided with alpha = 0.05.

## Results

### Demographic and diagnostic characteristics of the final sample

The HCL-33 was completed by 285 children and adolescents. Twelve children and adolescents (4.2%) left ≥ 3 questions unanswered, corresponding to > 10% of the 33 items. These individuals and their associated data did not differ significantly in terms of sex, age, IQ, or treatment setting (*p* = 0.23–0.96) from the final data set (n = 273) and were excluded from further analyses. In the final data set, we found that the items with the most frequent missing answers were in descending order: #7 (“tend to drive faster”) (n = 14, 5.1%), #33 (“eat more or binge more”) (n = 7, 2.6%), #16 (“more interested in sex”) (n = 6, 2.2%). All other items had ≤ 5 missing answers. Demographic and diagnostic characteristics of the final analyzed sample are presented in Table 1.

**Table 1:** Demographic and diagnostic characteristics

	Total sample (*n* = 273)	Female (*n* = 164)	Male (*n* = 109)	*p*-value
Demographic characteristics				
Age, years ± SD	15.0 ± 1.5 (range 12 − 18)	15.0 ± 1.5	14.9 ± 1.5	0.41
Sex, n (%)		164 (60.1)	109 (39.9)	**< 0.0001**
IQ^a^ ± SD	103.5 ± 13.3 (range 67 − 136)	103.2 ± 13.9	103.9 ± 12.8	0.65
Treatment setting, *n* (%)				
Outpatients	184 (67.4)	103 (62.8)	81 (74.3)	0.12
Inpatients	57 (20.9)	39 (23.8)	18 (16.5)	
Day-clinics	32 (11.7)	22 (13.4)	10 (9.2)	
Diagnostic characteristics, n (%)				
Mean number of diagnoses ± SD	1.1 ± 0.8	1.1 ± 0.8	1.1 ± 0.9	0.99
Adjustment disorders	74 (27.1)	44 (26.8)	30 (27.5)	1.00
Depressive disorders	72 (26.4)	51 (31.1)	21 (19.3)	**0.035**
ADHD	56 (20.5)	25 (15.2)	31 (28.4)	**0.009**
Anxiety disorders	39 (14.3)	22 (13.4)	17 (15.6)	0.72
Eating disorders	21 (7.7)	19 (11.6)	2 (1.8)	**0.002**
Schizophrenia-spectrum disorders	10 (3.7)	5 (3.0)	5 (4.6)	0.53
Conduct disorder	9 (3.3)	9 (5.5)	2 (1.8)	0.21
Substance use disorders	9 (3.3)	4 (2.4)	5 (4.6)	0.49
Autism-spectrum disorder	7 (2.6)	0 (0.0)	7 (6.4)	**0.001**
Personality disorders	5 (1.8)	3 (1.8)	2 (1.8)	1.00
Bipolar disorder	3 (1.1)	2 (1.2)	1 (0.9)	1.00
No diagnosis	55 (20.1)	29 (17.7)	26 (23.9)	0.22

*SD* standard deviation

^a^Data available for 167/273 (61.4%) of the total sample, for 88/164 (53.7%) females, 79/109 (72.5%) males, ADHD = Attention-Deficit-Hyperactivity-Disorder, group-differences were assessed using Mann–Whitney-U and bolded *p*-values represent significant effects

The mean age of the sample was 15.0 ± 1.5 (range 12.0–18.0) years. The majority was female (*n* = 164, 60.1%, *p* < 0.001). Mean IQ was 103.5 ± 13.3 (range 67–136). Most patients were treated in an outpatient setting (*n* = 184, 67.4%), 57 patients (20.9%) were treated in inpatient settings, and 32 (11.7%) in day-clinics. The most frequent clinical diagnoses among the young individuals were in descending order: adjustment disorders (*n* = 74, 27.1%), depressive disorders (*n* = 72, 26.4%), and ADHD (*n* = 56, 20.5%). Multiple diagnoses per patient were possible with a mean of 1.1 ± 0.8 (range: 0–4) diagnoses. Fifty-five individuals (20.1%) did not meet criteria for an ICD-10 diagnosis (Table 1). As the majority of the sample was female (60.9%), all demographic and diagnostic characteristics as well as the treatment setting were compared between female and male participants, revealing no differences for age, IQ, and treatment setting (Table 1). However, females were significantly more affected by depressive and eating disorders and less affected by ADHD and autism-spectrum disorders when compared to males (Table 1).

### (Hypo)manic symptoms and their associations with demographic and diagnostic characteristics

The mean frequency of affirmative responses on the HCL-33 in this sample was 14.7 ± 5.6, ranging between 0 and 29. HCL-33 sum score was not associated with sex and did not correlate with age or IQ. Regarding diagnostic groups according to ICD-10, the mean HCL-33 sum score was nominally highest in patients affected by depressive disorders (*n* = 72, 16.3 ± 5.6) and declining across patients with eating disorders (*n* = 21, 16.1 ± 5.1), anxiety disorders (*n* = 39, 14.3 ± 5.3), adjustment disorders (*n* = 74, 13.9 ± 5.4), ADHD (*n* = 56, 13.8 ± 6.3), and no confirmed psychiatric diagnosis (*n* = 55, 13.6 ± 5.5). HCL-33 sum scores were higher in patients with depressive disorders compared to help-seeking individuals without a mental disorder (16.3 ± 5.6 vs. 13.6 ± 5.5, *p* = 0.011). Excluding patients with depressive disorder and comorbid ADHD, patients with a depressive disorder (*n* = 66, 16.7 ± 5.4) showed higher HCL-33 sum scores compared to patients with ADHD (*n* = 50, 14.1 ± 6.3) (*p* = 0.036). Patients with ADHD or an anxiety disorder did not differ in HCL-33 sum scores compared to individuals with no confirmed psychiatric diagnosis (*p* = 0.91 or *p* = 0.96, respectively).

### Frequency of individuals at-risk for BD and group characteristics

80/273 (29.3%) had ≥ 18 affirmative responses in the HCL-33, and 162/267 (59.3%) had a CDI sum score ≥ 17; six individuals did not complete the CDI. The scatterplot in Fig. 1 illustrates the intersection of the respective groups.

In this analysis, children and adolescents were considered (1) “at-risk for BD” (*n* = 55; 20.6%), if they met both cut-off criteria for current depressive symptoms as well as cut-off criteria for lifetime (hypo)manic symptoms, and (2) “not at-risk for BD” (*n* = 107; 40.1%), if they only met the cut-off criteria for current depressive symptoms.

A group-level comparison showed that individuals in the at-risk for BD group were older (15.34 ± 1.47 vs. 14.81 ± 1.37, *p* = 0.030) and exhibited more anxious symptoms (*p* = 0.009) and conduct problems (*p* = 0.037), whereas increased hyperactivity did not reach the significance level (p = 0.061) (Table 2).

**Table 2:** Demographic characteristics and concurrent psychopathology in at-risk vs. not at-risk state for bipolar disorder

	Not at-risk for BD (CDI ≥ 17 and HCL-33 < 18) (*n* = 107)	At-risk for BD (CDI ≥ 17 and HCL-33 ≥ 18) (*n* = 55)	*p*-value
Sex, female; n (%)	71 (66.4%)	37 (67.3%)	1.00
IQ	104.79 ± 12.87^a^	104.39 ± 12.02^b^	0.72
Age, years	14.81 ± 1.37	15.34 ± 1.47	**0.030**
CDI sum score	24.41 ± 6.24	25.84 ± 5.79	0.075
HCL-33 sum score	12.64 ± 3.71	21.53 ± 2.34	**< 0.0001**
Anxious symptoms (BAI)	20.07 ± 12.11	25.00 ± 10.98	**0.009**
SDQ total score	17.05 ± 4.49	18.31 ± 4.69	0.074
SDQ emotional problems	5.74 ± 2.24	6.28 ± 2.12	0.17
SDQ conduct problems	2.43 ± 1.74	3.04 ± 1.84	**0.037**
SDQ hyperactivity	4.61 ± 2.00	5.26 ± 1.93	0.061
SDQ peer problems	4.28 ± 2.25	3.76 ± 2.36	0.20

*BAI* Beck Anxiety Inventory, *CDI* Depression Inventory for Children and Adolescents, *HCL* Hypomania Checklist, *SDQ* Strengths and Difficulties Questionnaire

All variables except sex are presented as mean ± SD, ^a^IQ available for n = 67, ^b^IQ available for *n* = 28, group differences were assessed using Mann–Whitney-U test and bolded *p*-values represent significant effects

**Fig. 2:**
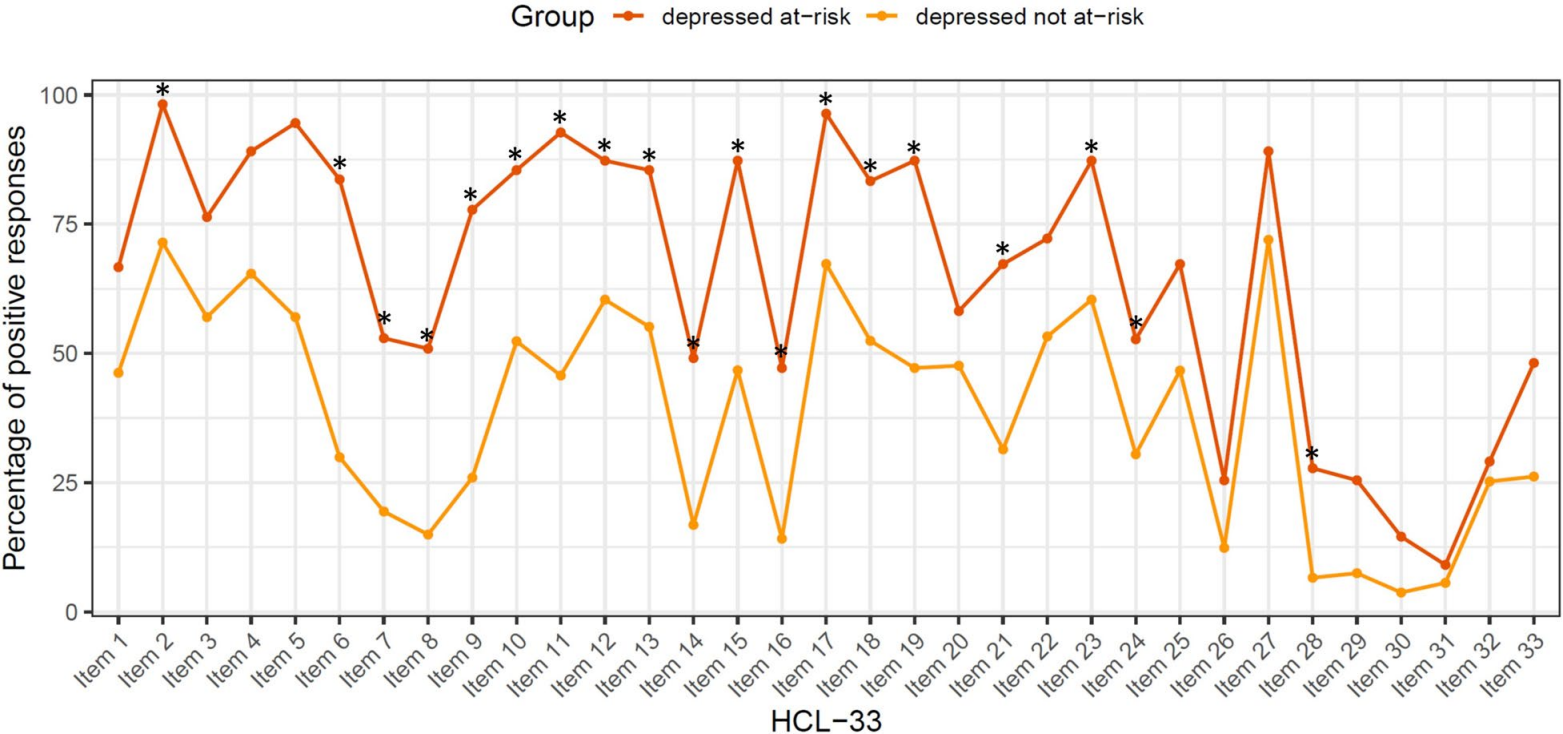
Percentage of positive responses for each HCL-33-item in at-risk vs. not at-risk state for BD. Percentage of affirmative responses in the HCL-33 comparing individuals with depressive symptoms at-risk for developing BD vs. not at-risk. Plotted on the y-axis are the 33 single items of the HCL-33. Plotted on the x-axis is the percentage of affirmative responses per item. The orange dots represent the corresponding percentages per item of the group not at-risk for developing BD (*n* = 107). The red dots represent the respective values of the group at-risk for developing BD (*n* = 55). Between-group differences were analyzed using a chi-square test and significant effects are marked with an asterisk after Bonferroni correction (33 performed tests per group, 0.05/33 = 0.0015)

Assessing the group-specific profile of the 33 items of the HCL-33, 19 items (# 2, 6–19, 21, 23, 24, 28) were more frequent in individuals in the at-risk for BD group (*p*-values below the value of 0.0015) (Fig. 2).

### Exploratory factor analysis

The Kaiser–Meyer–Olkin criterion of sampling adequacy was 0.8 and within the range of 0.61–0.90 for individual items, indicating the suitability of the data for factor analysis. Bartlett’s test of sphericity was significant [*χ*^*2*^(528) = 1652.28, *p* < 0.001], indicating that the observed correlation matrix was significantly different from the identity matrix, which further justifies the EFA procedure (Fig. 1S). The respective approaches to detect underlying factors yielded different potential factor solutions (Figs. 2S, 3S). We focussed on the three first factors of the seven-factor solution as neither statistical nor theoretical considerations support factors with ≤ 3 items as meaningfully interpretable (Fig. 4S). In addition, the VSS approach suggested a two-factor model, with VSS complexity 1 and 2 achieving maxima of 0.61 and 0.75, respectively (Fig. 5S). As both two- and three-factor solutions have been suggested for the HCL-32 and HCL-33, and as one finding based on a non-clinical population of adolescents suggested a third factor to be rather specific for this age group (Holtmann et al. 2009), both solutions were considered.

The factor loadings for the respective models are presented in Table 1S. The two-factor model consisted of Factor 1 (“active-elated”) comprising 11 items (# 2, 3, 4, 10, 11, 12, 13, 1, 17) and Factor 2 (“risk-taking/irritable”) comprising 17 items (# 5, 6, 7, 8, 9, 14, 16, 20, 21, 22, 24, 25, 26, 29, 30, 31, 33) (Table 1S). The three-factor-model included the same Factor 1. In contrast, Factor 2a (“irritable-erratic”) was composed of seven items (# 7, 9, 20, 22, 24, 25, 26) representing symptoms of irritability and erratic mood and the additional Factor 2b (“outgoing-disinhibited”) was composed of five items (# 5, 6, 14, 15, 16) representing outgoing to disinhibited activities. Six items (# 8, 21, 29, 30, 31, 33) of Factor 2 of the two-factor-model, were not included in the three-factor-model. Item # 15 (“I want to meet more people or I am doing it”) was included in the three-factor model and did not show relevant loading in the two-factor model. Cronbach’s alpha was 0.81 for all 33 items of the questionnaire. Likewise, the 11 items of Factor 1 yielded a Cronbach’s alpha of 0.81. Cronbach’s alpha was 0.76 for Factor 2 of the two-factor model and 0.71 and 0.65 for Factor 2a and 2b of the three-factor model, respectively.

### Associations of (hypo)manic sum and factor scores with demographic characteristics and current psychopathology

Whereas HCL-33 sum scores and Factor 2 and 2a were not associated with sex, age, or IQ, Factor 1 positively correlated with IQ (*r* = 0.16, *p* = 0.035) and Factor 2b positively correlated with age (*r* = 0.15, *p* = 0.016). Accordingly, in the following analyses, associations of the Factor 1 and Factor 2b were controlled for IQ or age, respectively.

The higher the HCL-33 sum and Factor 2 or 2a scores were, the higher were the concurrent depressive and anxious symptoms and the more severe were the SDQ total and the subscores “emotional problems”, “conduct problems”, and “hyperactivity” (Table 3). Higher Factor 1 (“active-elated”) scores were only associated with more severe SDQ emotional problems after Bonferroni correction for multiple comparisons. Higher Factor 2b (“outgoing-disinhibited”) scores were solely associated with more severe SDQ conduct problems (Table 3).

**Table 3:** Correlation analysis of hypomanic symptoms with current psychopathology

	HCL-33 sumscore		^a^HCL-33 Factor 1		HCL-33 Factor 2		HCL-33 Factor 2a		^b^HCL-33 Factor 2b	
	*r*	*p*	*R*	*p*	*r*	*p*	*r*	*P*	*r*	*p*
Depressive symptoms (CDI)	0.23	**< 0.001**	0.09	0.32	0.27	**< 0.001**	0.29	**< 0.001**	0.11	0.10
Anxious symptoms (BAI)	0.26	**< 0.001**	0.21	0.013	0.25	**< 0.001**	0.21	**< 0.001**	0.17	0.013
SDQ total score	0.29	**< 0.001**	0.20	0.021	0.38	**< 0.001**	0.40	**< 0.001**	0.10	0.15
SDQ emotional problems	0.21	**< 0.001**	0.24	**0.005**	0.18	**0.004**	0.20	**0.001**	0.07	0.32
SDQ conduct problems	0.30	**< 0.001**	0.00	1.00	0.44	**< 0.001**	0.42	**< 0.001**	0.20	**0.002**
SDQ hyperactivity	0.28	**< 0.001**	0.15	0.081	0.40	**< 0.001**	0.41	**< 0.001**	0.13	0.06
SDQ peer problems	− 0.027	0.66	0.06	0.48	0.01	0.93	0.05	0.40	− 0.11	0.10
SDQ prosocial behaviour	0.014	0.82	0.09	0.28	− 0.03	0.59	− 0.08	0.17	0.12	0.06

*BAI* Beck Anxiety Inventory, *CDI* Depression Inventory for Children and Adolescents, *HCL-33* 33-item Hypomania Checklist, *SDQ* Strengths and Difficulties Questionnaire

^a^HCL-33 Factor 1 adjusted for IQ, ^b^HCL-33 Factor 2b adjusted for age; bolded p-values represent significant findings of the correlations analyses after Bonferroni correction (8 performed tests per variable, 0.05/8 = 0.00625)

### Associations of (hypo)manic sum and factor scores with reported negative impact on four domains of daily life

Negative impact on one or more of the four domains of daily life (family life, social life, work/school, leisure) due to (hypo)manic symptoms was reported by 136/263 (51.7%) young individuals. The frequency of individuals reporting negative impact varied depending on the distinct domain of daily life. The highest number of individuals reported negative impact of their (hypo)manic symptoms on family life (*n* = 91, 34.0%), followed by work/school life (*n* = 78, 30.8%). Only a minority reported negative impact on leisure (*n* = 45, 16.8%) or social life (*n* = 43, 16.1%).

The associations of HCL-33 sum and factor scores with reported negative impact on the four domains of daily life are presented in Table 4. Compared to individuals who do not report negative impact, individuals experiencing negative impact of their hypomanic symptoms on work/school life, show higher HCL-33 sum and Factor 2, 2a, and 2b scores. Further, children and adolescents who reported negative impact on leisure, family and social life showed significantly higher HCL Factor 2 and 2a scores. In contrast, individuals who did not report negative impact on their family life, showed higher HCL-33 Factor 1 scores (Table 4).

**Table 4:** Comparison of HCL-33 scores between individuals affirming vs. individuals denying negative impact on daily life

	Negative impact on											
	Family life (*n* = 268)			Social life (*n* = 267)			Work/school (*n* = 253)			Leisure (*n* = 268)		
	Yes (*n* = 91)	No (*n* = 177)	*p*-value	Yes (*n* = 43)	No (*n* = 224)	*p*-value	Yes (*n* = 78)	No (n = 175)	*p*-value	Yes (*n* = 45)	No (*n* = 223)	*p*-value
HCL-33 sum score	15.1 ± 5.6	14.6 ± 5.6	0.80	16.1 ± 6.0	14.6 ± 5.4	0.23	16.7 ± 5.3	14.2 ± 5.4	**0.002**	15.7 ± 5.7	14.6 ± 5.5	0.25
HCL-33 F.1	6.8 ± 3.0	7.9 ± 2.9	**0.002**	7.1 ± 3.0	7.6 ± 3.0	0.26	7.5 ± 2.9	7.7 ± 2.9	0.49	7.0 ± 3.1	7.6 ± 3.0	0.23
HCL-33 F.2	6.3 ± 3.4	4.8 ± 3.2	**0.001**	7.1 ± 3.6	4.9 ± 3.2	**< 0.001**	6.9 ± 3.5	4.6 ± 3.1	**< 0.001**	6.8 ± 3.4	5.0 ± 3.3	**0.002**
HCL-33 F.2a	3.1 ± 1.9	2.2 ± 1.9	**< 0.001**	3.8 ± 1.9	2.2 ± 1.9	**< 0.001**	3.4 ± 2.0	2.0 ± 1.8	**< 0.001**	3.8 ± 1.7	2.2 ± 1.9	**< 0.001**
HCL-33 F.2b	2.4 ± 1.5	2.0 ± 1.5	0.061	2.3 ± 1.6	2.2 ± 1.5	0.63	2.5 ± 1.5	2.0 ± 1.5	**0.012**	2.3 ± 1.5	2.1 ± 1.5	0.49

*HCL-33* Hypomania Checklist-33, *HCL-33 F* HCL-33 Factor

Group-differences were assessed using Mann–Whitney-U test and bolded *p*-values represent significant findings after Bonferroni correction (4 performed tests per variable; 0.05/4 = 0.0125)

## Discussion

The present study investigated self-reported lifetime (hypo)manic symptoms and associations with current diagnostic and psychopathological characteristics in children and adolescents from a mixed psychiatric sample. Applying the combined risk criteria of ≥ 18 (hypo)manic symptoms lifetime as well as current depressive symptoms, 20.6% (*n* = 55) of the total sample was classified as at-risk for BD. At-risk individuals were older and reported more anxious symptoms and conduct problems when compared to individuals in the not at-risk for BD group. Statistical and theoretical considerations supported a two- and a three-factor structure of the HCL-33 as most adequately representing the symptom dimensions of (hypo)mania in this sample of children and adolescents. The HCL-33 sum and factor scores showed distinct associations with current psychopathology as well as with reported negative impact on daily life.

### At-risk grouping or “missed” bipolar-spectrum diagnoses

Fifty-five individuals (20.6%) were detected as at-risk for BD using the combined risk criteria of current depressive symptoms and lifetime (hypo)manic symptoms. This number may seem high, especially in contrast to the number of individuals who met BD criteria according to clinical ICD-10 diagnosis (n = 3, 1.1.% of the total sample).

While according to more restrictive definitions only 1.8% of the general population meet criteria for BD, 16–21% of referrals to mental health services for children and adolescents or patients treated in such clinics meet diagnostic criteria for BD (Biederman et al. 2005; Meter et al. 2019; Weller et al. 1995). A previous study identified that 16% of children and adolescents referred to a child psychiatry service (*n* = 1838) met criteria for BD using intensive structured diagnostical interviews with the child and caregiver, as well as consensus conferences on the diagnosis (Biederman et al. 2005). Thus, the introduction of screening for (hypo)manic symptoms in routine diagnostics, especially in children and adolescents with current depressive symptoms may be time-effective and suitable to identify patients who need more in-depth diagnostics. An approach to increase the specificity of the screening may be to carefully introduce the HCL during an appointment and guide the identification of a potential “high” phase in the past as previously carried out in one longitudinal study in adolescents of the general population (Nielsen et al. 2021). This approach may also reduce the number of false-positive at-risk individuals due to potential affirmative items related to phases under the influences of psychoactive substances or early romantic love (Brand et al. 2007; Marwaha et al. 2018; Nielsen et al. 2021). Moreover, especially for young individuals of the general population, cannabis use seems to drive an increased frequency of reported hypomanic symptoms according to the HCL (Marwaha et al. 2018; Nielsen et al. 2021). Lacking specific questionnaires to screen for type, frequency and severity of intake of psychoactive substances, it was not possible to control for this factor in this mixed clinical sample.

In another approach, this study also detected and compared the frequency of (hypo)manic symptoms in patient groups defined by their clinical ICD-10 diagnosis. Interestingly, the patient group affected by depressive disorders reported a mean frequency of 16.3 (hypo)manic symptoms in the HLC-33 corresponding to the mean previously found in adolescents diagnosed with BD (16.6) and corresponding to the mean range found in adults diagnosed with BD (16.2–21.3) as assessed with the HCL-32 or HCL-33 (Angst et al. 2005; Forty et al. 2009; Feng et al. 2016; Meyer et al. 2011; Rybakowski et al. 2012; Zhang et al. 2021). These findings support the assumption that there are potentially “missed” bipolar-spectrum diagnoses in this mixed sample, especially among the at-risk for BD group or among patients clinically identified as currently affected by a depressive disorder.

Compared to individuals in the not at-risk for BP group, the at-risk group was significantly older and experienced more severe anxious symptoms and conduct problems. These group-specific differences in self-reported symptomatology seem to correspond to a high rate of anxious symptoms preceding BD and to the high comorbidity of BD with anxiety (53–90%) and conduct disorders (36–48%) (Biederman et al. 2005; Faedda et al. 2014; McIntyre et al. 2020). Self-reported symptoms of hyperactivity were not significantly higher in the at-risk for BD group compared to the not at-risk for BD group but reached trend level significance (*p* = 0.061). It remains unclear to what extent this could be influenced by the overall high rate of patients clinically diagnosed with ADHD (20.5%) in the total sample.

Focusing on patients clinically diagnosed with ADHD, the self-reported mean frequency of (hypo)manic symptoms in the HCL-33 was comparable to individuals with no diagnosed mental disorder (13.8 vs. 13.6). This was unexpected to a certain extent, as there is a high comorbidity (25–45%) of adults with ADHD and BP, and this comorbidity seems to be driven by patients with onset of the disorder before age 18 (McIntyre et al. 2020; Lan et al. 2015; Sachs et al. 2000). In view of moderate group sizes, this finding has to be interpreted with caution. The entanglement of (hypo)manic symptoms with symptoms of ADHD, especially in prepubertal children, has been at the heart of critical discussions regarding discriminatory aspects of those diagnoses ( Geller et al. 1998; Perlis et al. 2004; Weller et al. 1995; Wozniak et al. 2004). In this study, the self-reports were collected in a mixed psychiatric sample of 12–18-year-olds, limiting the ability to disentangle the specifics of prepubertal children, ADHD, and (hypo)manic symptoms. Likewise, it may also be particularly difficult for young patients with a clinical diagnosis of ADHD to report lifetime (hypo)manic symptoms in a self-report. First studies implemented a version of the HCL-33 for caregivers (Chen et al. 2022; Wang et al. 2021). It may be of interest to assess the value of the external observation when differentiating between ADHD and BD, especially in younger children. Longitudinal studies will be needed to investigate the predictive validity of the HCL-33 in different age group samples.

### Factor structure and associations with current psychopathology and with reported negative impact on daily life

Parallel to previous studies either using the HCL-33 in a clinical, adolescent sample, or using the HCL-32 in clinical and non-clinical, adult samples, this work showed that the HCL-33 items load on two factors representing the “bright” and the “dark” side of mania (Brand et al. 2011; Gamma et al. 2013; Hantouche et al. 2003; Meyer et al. 2014). In addition, a three-factor solution was pursued. Interestingly, the “bright” or “active-elated” Factor 1 was similar in both factor solutions and only showed a positive correlation with emotional problems. A study in adolescents of the general population also found a three-factor solution. However, they reported no association of Factor 1 with emotional problems or other current psychopathology, but rather a negative correlation with peer problems as assessed with the SDQ, which was also used in our study (Holtmann et al. 2009). Further, individuals who did not report any negative impact of (hypo)manic symptoms on their family life had higher Factor 1 scores compared to individuals who reported negative impact. This finding may contribute to the fact that neither the adolescents nor their families are prompted to seek help in the context of (hypo)manic symptoms belonging to the “bright” side.

Pursuing the three-factor solution, items of the “dark” side also loaded on two separate factors, “irritable-erratic” (2a) and “outgoing-disinhibited” (2b). Higher Factor 2a scores were associated with more severe symptomatology across several symptom domains and were found in individuals reporting negative impact in all four domains of daily life. Higher Factor 2b scores correlated only with more severe conduct problems and were found in individuals reporting negative impact on their work/school life. Direct comparisons with previous studies are limited because of the use of the former version of the HCL, but in general, the findings correspond to the three-factor solution in healthy adolescents with a similar Factor 2a that correlated with SDQ total difficulties score and several subscores, and a similar Factor 2b, which was correlated especially with conduct problems (Holtmann et al. 2009). Taken together, the findings point to specific psychopathological correlates of the “bright” and the “dark” side of hypomanic symptomatology in a mixed paediatric psychiatric sample.

### Limitations

The findings from this study should be considered in the context of its limitations. First, data were collected in a single mental health service and standardized diagnostical interviews were lacking. Therefore, generalizability and comparability are limited. Second, while the amount of questionnaires admininstered in each clinic appears to be representative of the care structure, the treatment setting and severity of overall illness of participants may substantially impact findings on dimensionality and associations of (hypo)manic symptoms. This should be considered when comparing findings to outcomes in other settings Third, we used the BAI for the self-assessment of anxiety symptoms in 12–18-year-old, although the BAI has been used and validated mostly in adolescents with an age-range 14–18 (Jolly et al. 1993; Osman et al. 2002). For the adolescents ages 12 and 13, this may have led to less than optimal understanding of all items. Fourth, providing answers to the 33 symptom items on the HCL-33 did seem feasible for children and adolescents in this age range. Hence, a small number of missing answers occurred in those items asking for behaviour that may not be applicable to or less of an issue for younger individuals (i.e., risky driving, sexual desire). This is in line with the pattern of missing answers found in an older sample of adolescents and young adults (15.3–20.4 years) (Holtmann et al. 2009). Thus, younger individuals may need help to address these questions or adaptions in language or the description of the symptom may be necessary in future studies in young individuals. Fifth, the focus on patients meeting the combined risk-criteria does not take into account a significant proportion of young individuals who experience (hypo)mania as their first episode of BD without depressive symptoms (Hauser and Correll 2013). Sixth, we did not employ specific questionnaires to screen for type, frequency and severity of intake of psychoactive substances including illegal drugs. Since substance use can affect mood and be associated with (hypo)mania-like symptomatology, studies focusing on BD risk groups should specifically measure degree of substance use and, ideally, separate symptoms that occurred during versus without substance use (Marwaha et al. 2018; Tijssen et al. 2010). Finally, the cross-sectional design cannot discern missed diagnoses of BD from individuals at-risk for developing BD or calculate the predictive validity of the at-risk status.

## Conclusions

The high frequency of reported (hypo)manic symptoms as well as the high percentage of individuals with increased risk for BD support the notion that a careful assessment of lifetime (hypo)manic symptoms is needed in children and adolescents presenting with depressive and/or a broad range of symptomatology in order not to miss a BD diagnosis. The short HCL-33 may support routine screening and prompt in-depth diagnostic efforts in mixed paediatric psychiatric samples to detect individuals who experience (hypo)manic episodes. Longitudinal studies are needed to advance and refine our understanding of the chronology and phenomenology of the symptomatology, the at-risk state for BD as well as protective factors in children and adolescents (Bechdolf et al. 2012b; Faedda et al. 2019; Hauser and Correll 2013; Hafeman et al. 2016; Leopold et al. 2014).

### Supplementary Information

Supplementary Material 1.

## Data Availability

The dataset used and/or analysed during the current study are available from the corresponding author on reasonable request.

## Abbreviations

ADHD: Attention-Deficit-Hyperactivity Disorder
BAI: Beck Anxiety Inventory
BD: Bipolar Disorder
CDI: Depression Inventory for Children and Adolescents
EFA: Exploratory factor analysis
HCL-32/33: 32-Item or 33-item Hypomania Checklist
ICD-10: International Classification of Diseases-10
IQ: Intelligence quotient
KEK-ZH: Local ethics committee of the Canton Zurich
MDD: Major Depressive Disorder
SD: Standard deviation
SDQ: Strengths and Difficulties Questionnaire
VSS: Very Simple Structure
