# Supplementary material for: Hypomania-Checklist-33: risk stratification and factor structure in a mixed psychiatric adolescent sample
Source: Int J Bipolar Disord. 2024 Aug 7;12:28. doi: 10.1186/s40345-024-00350-x (PMC11306698; doi:10.1186/s40345-024-00350-x)
Supplement: Supplementary file 1 — Supplementary Material 1. [file 40345_2024_350_MOESM1_ESM.docx]

**Supplementary material**

**Table 1S: Two-factor- and 3-factor-model and respective factor loadings**

|  | ***n*=220** | **HCL-33 two-factor solution**  **(VSS)** | | **HCL-33 three-factor solution**  **(parallel analysis)** | | |
| --- | --- | --- | --- | --- | --- | --- |
|  |  | Factor 1  Active-elated | Factor 2  Risk-taking-irritable | Factor 1  Active-elated | Factor 2a  Irritable-erratic | Factor 2b  Outgoing-disinhibited |
| 1 | I need less sleep |  |  |  |  |  |
| 2 | I feel more energetic and more active | 0.85 |  | 0.83 |  |  |
| 3 | I am more self-confident | 0.77 |  | 0.77 |  |  |
| 4 | I enjoy my work more | 0.62 |  | 0.67 |  |  |
| 5 | I am more sociable (make more phone calls, go out more) |  | 0.44 |  |  | 0.78 |
| 6 | I want to travel and/or do travel more |  | 0.57 |  |  | 0.50 |
| 7 | I tend to drive faster or take more risks when driving |  | 0.63 |  | 0.40 |  |
| 8 | I spend more money/too much money |  | 0.47 |  |  |  |
| 9 | I take more risks in my daily life (in my work and/or other activities) |  | 0.63 |  | 0.45 |  |
| 10 | I am physically more active (sport etc.) | 0.49 |  | 0.42 |  |  |
| 11 | I plan more activities or projects | 0.46 |  | 0.57 |  |  |
| 12 | I have more ideas, I am more creative | 0.63 |  | 0.61 |  |  |
| 13 | I am less shy or inhibited | 0.54 |  | 0.62 |  |  |
| 14 | I wear more colourful and more extravagant clothes/make-up |  | 0.48 |  |  | 0.46 |
| 15 | I want to meet or actually do meet more people |  |  |  |  | 0.79 |
| 16 | I am more interested in sex and/or I am more sexually active |  | 0.46 |  |  | 0.73 |
| 17 | I talk more | 0.68 |  | 0.56 |  |  |
| 18 | I think faster | 0.53 |  | 0.66 |  |  |
| 19 | I make more jokes or puns when I am talking |  |  |  |  |  |
| 20 | I am more easily distracted |  | 0.46 |  | 0.62 |  |
| 21 | I engage in lots of new things |  | 0.59 |  |  |  |
| 22 | My thoughts jump from topic to topic |  | 0.65 |  | 0.61 |  |
| 23 | I do things more quickly and/or more easily | 0.76 |  | 0.58 |  |  |
| 24 | I am more impatient and/or get irritable more easily |  | 0.60 |  | 0.85 |  |
| 25 | I can be exhausting or irritating for others |  | 0.48 |  | 0.75 |  |
| 26 | I get into more quarrels |  | 0.56 |  | 0.63 |  |
| 27 | My mood is higher, more optimistic | 0.85 |  | 0.69 |  |  |
| 28 | I drink more coffee |  |  |  |  |  |
| 29 | I smoke more cigarettes |  | 0.55 |  |  |  |
| 30 | I drink more alcohol |  | 0.54 |  |  |  |
| 31 | I take more drugs (sedatives, anxiolytics, stimulants...) |  | 0.41 |  |  |  |
| 32 | I game or gamble more |  |  |  |  |  |
| 33 | I eat more or I binge more |  | 0.46 |  |  |  |
|  | Items included in the respective factore | 11 | 17 | 11 | 7 | 5 |

Abbreviation: HCL-33, 33-item Hypomania Checklist.

**Fig. 1S: Tetrachoric correlation matrix of the HCL-33 data.**

**Fig. 2S: Scree plot depicting the eigenvalues of factors as a function of factor number.**

**Fig. 3S: Graphical output of the parallel analysis.**


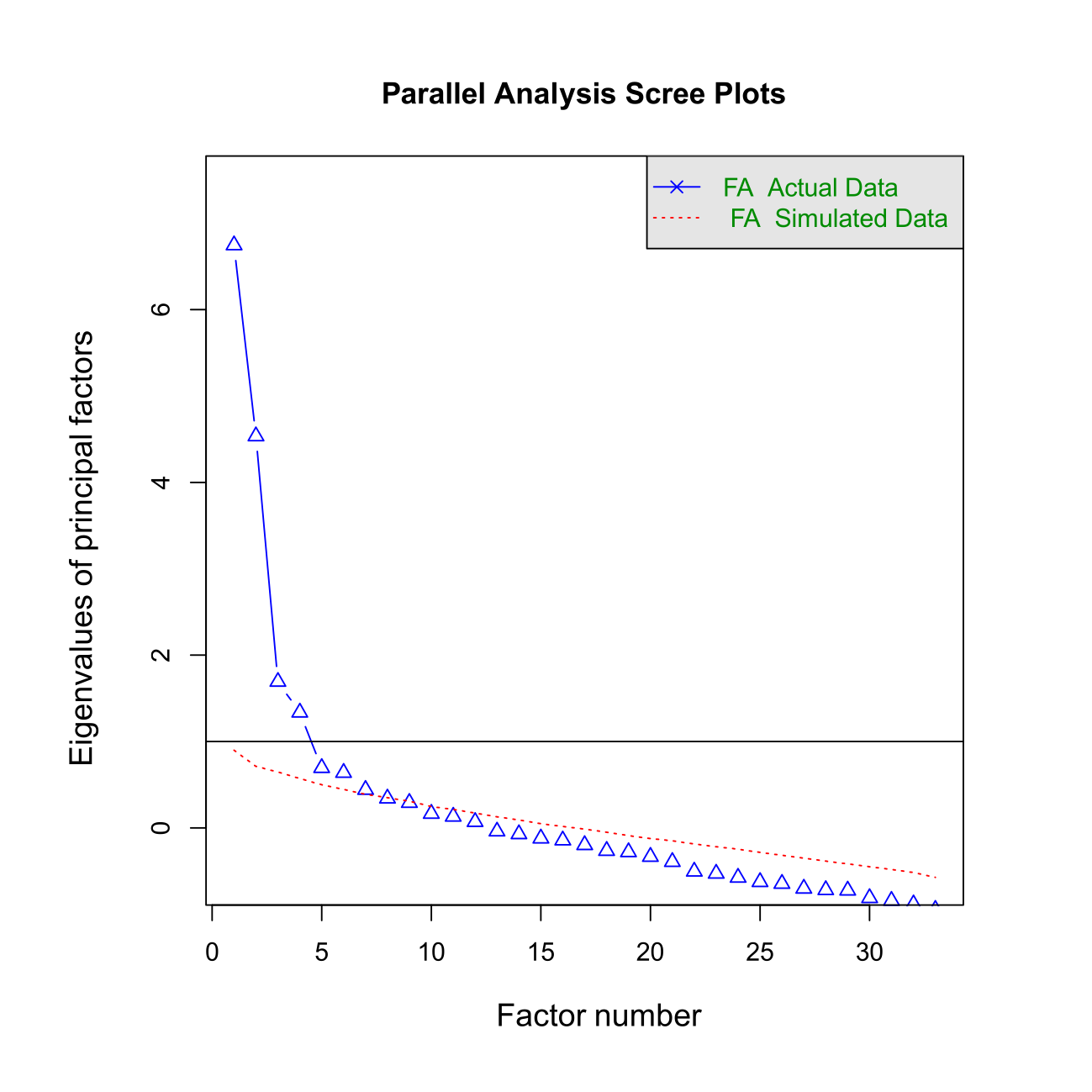


The red line depicts the simulated eigenvalues, while the blue triangles depict the eigenvalues of potential factors in the observed data.

**Fig. 4S: Graphical representation of the seven-factor solution, as indicated by the parallel analysis.**

**Fig. 5S: Model fit as a function of factor number, as indicated by the Very Simple Structure.**
